# Bridging the AI-Literacy Gap in Health Care: Qualitative Analysis of the Flanders Case Study

**DOI:** 10.2196/76709

**Published:** 2025-12-08

**Authors:** Christos Chatzichristos, Georgios Chatzichristos, Isabelle Borremans, Stefaan Gruyaert, Ilse De Vos, Maarten De Vos, Femke De Backere

**Affiliations:** 1 Department of Electrical Engineering, STADIUS Center for Dynamical Systems, Signal Processing, and Data Analytics, KU Leuven, Kasteelpark Arenberg 10, Leuven, 3001, Belgium, 32 0456087126; 2 Vlaamse AI Academie (VAIA), Belgium; 3 School of Political Sciences, Aristotle University of Thessaloniki, Thessaloniki, Greece; 4 Department of Development and Regeneration, KU Leuven, Leuven, Belgium; 5 IDLab, Department of Information Technology, Ghent University, Ghent, Belgium

**Keywords:** AI literacy, cross-disciplinary collaboration, occupational divides, structural inequalities, tailored training, lifelong learning, upskilling

## Abstract

**Background:**

Building on the assertion that nearly every clinician will eventually use artificial intelligence (AI), this study provides a triangulated qualitative analysis of the requirements, challenges, and prospects for integrating AI into routine health care practice. This skills gap contributes to cautious and uneven adoption across clinical settings. Despite advancements, many health care professionals report a self-perceived lack of proficiency in comprehending, critically evaluating, and ethically deploying AI tools, which contributes to cautious adoption in clinical settings.

**Objective:**

While addressing key research questions, the study investigates the necessary prerequisites, barriers, and opportunities for AI adoption and specific training priorities that medical staff require. The study is uniquely focused on the health care workforce, moving beyond the predominant emphasis in the literature on medical students.

**Methods:**

Situated in Flanders, Belgium, a recognized innovation leader but with moderate lifelong learning participation, this research combines 15 semistructured expert interviews, a regional survey of 134 health care professionals, and 3 co-interpretive focus groups with 39 stakeholders, all conducted in 2024.

**Results:**

The results expose small generational and mainly occupational divides. For instance, 85.07% (114/134) of survey respondents expressed interest in introductory AI courses tailored to health care, while 80% (107/134) of them sought practical, job-relevant AI skills. However, only 13.8% (19/134) of clinicians felt that their training adequately prepared them for AI integration. Notably, younger professionals (<30 years of age) were most eager to engage with AI but also expressed greater concern about job displacement, while older professionals (>50 years of age) prioritized reducing administrative burden. Physicians and dentists reported higher self-assessed AI knowledge, whereas nurses and physiotherapists showed the lowest familiarity. The survey also revealed differences in preferred learning formats, with doctors favoring flexible, asynchronous learning and nurses emphasizing the need for accredited, employer-supported training during work hours. Ethics, though emphasized in academic literature, ranked low in training interest among most practitioners, except for younger and palliative care professionals. Focus group participants confirmed the need for clear regulatory guidance and access to accredited, practically oriented training. A significant insight was that nurses often lacked institutional support and funding for training, despite their pivotal role in AI-enabled workflows.

**Conclusions:**

Taken together, these findings indicate that a one-size-fits-all approach to AI education in health care is unlikely to be effective. By triangulating insights across research stages, this study highlights the need for occupation-specific, accessible, and accredited AI training programs that bridge gaps in digital literacy and align with practical clinical priorities. The qualitative insights obtained can inform policy and training priorities in light of the European Union (EU) AI literacy mandates, while highlighting persistent gaps in workforce preparation.

## Introduction

The Information Age has spurred health care innovation by providing extensive data access and technology during the last 40 years, empowering the workforce to revolutionize health care. The emergence of artificial intelligence (AI) stands out as one of the most transformative elements of this era. Amid present conditions, AI technologies are increasingly becoming prominent in the analysis of diverse health data [1]. They are accelerating the processes of diagnosis and therapy, enhancing imaging techniques, providing guidance for surgical procedures, and streamlining drug research, thereby enabling the development of more personalized therapies [2].

In health care, AI has already shown remarkable promise, from analyzing chest X-rays and skin lesions (Keane and Topol [3]) to increasing understanding of individual treatment response for COPD [4] and significantly decreasing the time needed to review seizure events [5,6]. Despite these advancements, scaling AI faces hurdles, such as technical limitations, trust deficits, privacy concerns, algorithmic biases, and systemic issues, such as misaligned incentives, fragmented workflows [7], or even lack of more general, basic data and AI literacy among health care professionals. Moreover, recent studies and frontline experiences reveal that stakeholder resistance (driven by concerns about professional autonomy, patient safety, and the opaque nature of certain AI systems) remains a substantial and persistent challenge [8]. Regulatory frameworks remain in flux, and despite the European Union Artificial Intelligence (EU AI) Act and other policy initiatives, many clinical environments still grapple with gaps in guidance, accountability, and the standardization of ethical and safety protocols. These realities contribute to a landscape where the transformative promises of AI are frequently counterbalanced by practical and institutional constraints, underscoring the need for nuanced, context-specific analyses of implementation [9]. Addressing these challenges requires a strategic approach that emphasizes investments in human capital, clear regulatory frameworks, and improved data and AI literacy. To overcome these barriers, a human-centered design approach and revised frameworks tailored to health care–specific challenges are essential [10].

One increasingly acknowledged, yet still underresearched, area where AI could provide significant support is the reduction of administrative burden in health care. Studies suggest that clinicians spend up to half of their working hours on documentation, scheduling, and other nonclinical tasks, leading to burnout and reduced time with patients [11]. AI-driven solutions, such as automated note-taking, intelligent triage systems, and smart scheduling assistants, hold promise in alleviating this load. However, while the literature often emphasizes diagnostic and clinical decision-making applications of AI, the administrative potential of AI remains relatively underexplored in both academic and policy discussions. This gap limits the development of training programs and tools that target what are likely among the most immediate and practical benefits of AI for the existing workforce.

Although the usage of AI tools in clinical practice is on the rise, there exists a self-reported deficiency in the ability to comprehend, critically evaluate, and ethically use these tools in everyday clinical settings [12]. As a result, there is still an apparent reluctance by the medical workforce to use AI clinically [13]. Given these contexts, an increasing number of researchers and organizations underscore the importance of integrating AI into medical education, residency training, and continuous education programs for medical staff.

There is a well-documented gap in postgraduate and lifelong learning opportunities for health care professionals to acquire AI-specific skills, particularly compared to students [14]. Systematic reviews have found that most AI training initiatives target medical students or early-career phases, while working professionals (especially nurses and allied staff) must often seek out fragmented or informal learning, with formal, occupation-relevant programs remaining notably rare [15]. While AI-related continuing education has expanded in theory, actual uptake remains low due to lack of system-wide support, limited funding, and time constraints among health care staff, as reported in a very recent Lancet publication [16]. This literature underscores that competency gaps in areas such as algorithm appraisal, ethical and legal application, and digital literacy persist well after graduation, risking uneven and sometimes unsafe AI adoption in clinical practice. These findings demonstrate the urgency and relevance of research focused directly on health care workers’ lifelong learning needs and AI upskilling beyond academic education.

A survey found that only 13.8% of clinicians believed their training programs adequately prepared them for integrating AI into their practice [3]. However, another survey [17] shows that 71% of clinicians in ophthalmology, radiology, and dermatology reported optimism about AI improving their fields, particularly in disease screening and reducing monotonous tasks. This indicates that expectations for AI solutions are high among health care professionals. However, 80.9% had never used AI in practice, and only 5.5% rated their knowledge as “excellent.” Interestingly, ophthalmologists reported the highest usage of AI (15.7%), compared to radiologists (6.1%) and dermatologists (5.2%), highlighting disparities in AI adoption between occupations that reflect differences in exposure and training [17]. Significant divergences are also identifiable between different generations. Clinicians with less than 5 years of experience were twice as likely to perceive AI’s impact as profound compared to those with more than 30 years of practice. This generational divide underscores the need for tailored strategies to foster acceptance and implementation.

The successful integration of AI into health care systems faces fundamental challenges, including user acceptance, consistent usage in day-to-day operations, and seamless integration into the broader clinical workspace [18]. Overcoming these hurdles is essential to ensure that AI technologies become an integral and widely accepted part of routine medical practice. As in other nonmedical professions, guidelines emphasize that training related to AI is crucial for its effective integration into everyday practice [19], not only within preregistration health care programs but also as a means of continuously developing the skill set of the existing workforce. While a growing volume of literature advocates for the inclusion of AI-related content in medical curricula [20,21], the studies examining the training needs of the medical workforce in relation to AI are relatively scarce [13].

In line with these considerations, this study explores the following research questions:

1. What are the requirements, obstacles, and prospects for medical staff in integrating AI into their daily tasks?
2. What are their key priorities and needs for AI training in these areas?

To address these questions, the study conducts a thorough needs assessment of the overall medical workforce [22]. The studies that have explored the AI training among the medical workforce have mostly concentrated on specific specializations, such as clinical imaging [13] or electrocardiogram interpretation [23]. Few holistic approaches that explore the large-scale AI deployment in health care practices call for investments in human capital, clear regulations, and enhanced AI literacy [7].

The study leverages existing literature to implement multistage, qualitative research focusing on the health care professionals in the Flemish region of Belgium, a region that is characterized as an innovation leader and reflected in a comprehensive AI policy plan [24], but showing only moderate achievements in lifelong learning [25]. Accordingly, this focused investigation holds promise for enriching existing literature. The aim of this study is to systematically assess the prerequisites, barriers, and opportunities for AI adoption in health care, with a specific focus on identifying the training needs of the existing health care workforce in Flanders. Foregrounding AI literacy gaps within the health care workforce is particularly urgent, as the forthcoming EU AI Act will impose new obligations on providers and institutions to ensure safe, equitable, and competent AI adoption. The rest of the study is structured as follows. The “Methods” section delves into the relevant literature for the adoption of AI into Flanders and integration of AI training in the health care systems and presents the design and methodology of this study. The “Results” section illuminates the results from our research, while the “Discussion” draws insights from the results and discusses limitations and pathways for future research.

## Methods

### Learning Needs for AI in Health Care

An international survey [26] of 4596 medical, dentistry, and veterinary students from 48 countries found that 67.6% (n=3091) have positive attitudes toward AI in health care, and 76.1% (n=3474) desire more AI education in their curricula. Despite this interest, 75.3% (n=3451) reported limited general knowledge of AI and indicated the absence of AI-related courses in their programs. Additionally, most respondents felt unprepared to use AI in their future careers, underscoring the necessity for curriculum enhancements to include AI competencies. The receptiveness of medical and dental students to AI applications is particularly indicative of how AI can be integrated into graduate training, preparing the next generation of health care professionals for a future deeply intertwined with technological advancements. Cross-sectional studies, involving 3018 medical students across Turkey, further emphasized concerns about potential job reductions due to AI (44.9%), and the fear of AI devaluing the medical profession (1769, 58.6%). Along these lines, notably, only 6% (n=181) of the respondents felt competent to inform patients about AI’s features and risks, highlighting a significant literacy gap. In this vein, the majority expressed a need for training in AI applications, error reduction, and ethical problem-solving related to AI use [27].

Similar studies (with smaller samples) confirm these findings and further underscore the necessity of incorporating practical and ethical AI training into medical education to prepare students for the realities of AI-supported health care [28]. Furthermore, experts emphasized the importance of integrating ethical considerations into AI education for medical students [29]. In this regard, they advocated for a curriculum that balances technical AI skills with discussions on ethical implications, ensuring that future physicians can navigate the complexities of AI applications responsibly. This approach aims to foster a generation of health care professionals who are not only proficient in AI technologies but also mindful of their ethical responsibilities.

All the above findings indicate a pressing need to update medical education curricula to include comprehensive AI training [3]. Such training should cover practical AI applications, ethical considerations, and strategies to mitigate potential negative impacts on the medical profession. By addressing these educational gaps, future health care professionals can be better equipped to integrate AI into their practice, ultimately enhancing patient care and maintaining the integrity of the profession. Moreover, medical education serves as the essential foundation for the entire continuum of health care workers’ training. Refined curricula should also consider inconsistencies in AI training across institutions, highlighting the challenges of standardizing education systems globally [30].

### Contextualizing the Study: An Outline of Flanders’ Health Care

This study explores the integration of AI in health care training in Flanders, a region of Belgium (NUTS1 [Nomenclature des Unités Territoriales Statistiques] statistical region-Nomenclature of territorial units for statistics), which consists of 5 NUTS2 subregions, that is, Antwerp (BE21), Limburg (BE22), East Flanders (BE23), Flemish Brabant (BE24), and West Flanders (BE25). The health care system in Flanders is technologically advanced and operates under a mixed public-private model, offering universal coverage to residents. A network of hospitals, general practitioners, and specialists provides the care, which is mostly funded by social security payments. Primary care and preventative health services are highly valued in Flanders, with an emphasis on patient choice and accessibility.

Flanders’ health care system, although resource-rich and technologically advanced, is facing challenges due to an aging workforce that extends throughout the Belgian health care system. More analytically, Belgium’s health care workforce is aging more rapidly compared to the EU average. The percentage of practicing physicians in Belgium aged 55 years or older was 24.1% in 2000, and this percentage increased rapidly to 44.9% in 2016, surpassing the EU-12 average of 34.5% for the same year. Most recent data indicate that this trend continues, as in 2020 the percentage of Belgian physicians aged 55 years and older was 43.3%, surpassing the EU-14 average of 35.1% and the EU-27 average of 37.4%. Against this background, several key specialties have an even higher proportion of aging practitioners, such as rheumatologists (46.1%), general practitioners (44.6%), and radiologists (41%). This aging workforce exacerbates existing shortages, straining the system’s ability to meet the growing health care demands of an aging population. Despite efforts to address the issue, the percentage of older physicians in Belgium remains significantly higher than the EU-27 averages, posing a long-term challenge for health care sustainability [31].

Indeed, while AI is rapidly being adopted across the global economy, Flanders remains behind in this transformative shift. As of early 2024, an impressive 72% of companies worldwide reported using AI in at least one aspect of their operations [32]. However, a more recent study (conducted in 2023) from Flanders indicates that only 32.1% of companies have embraced AI within their business processes [33]. This difference underscores a need for greater awareness and integration of AI technologies in the region. One of the key challenges in Flanders is a lack of perceived necessity. A substantial 65.5% of Flemish companies believe that AI offers no significant benefits to their business. Compounding this issue, 64.5% admit to lacking the requisite knowledge, skills, and experience to effectively implement AI solutions. These barriers highlight an urgent need for targeted education and skill-building initiatives to bridge the gap between potential and practice. Although Flanders is recognized as a European innovation leader, it achieves only the European average when it comes to lifelong learning participation [25]. This was further confirmed by the Monitoring Report from the Flemish Department of Work and Social Economy [34]. As the workplace continues to evolve, 95% of employers anticipate that the skills required of professionals will change significantly in the coming years. However, this shift is met with resistance, as 40.7% of adults in Flanders currently do not participate in any form of training and express no interest in doing so, as they see no need for learning.

When asked about lifelong learning specifically related to AI, 44% of European respondents expressed skepticism, stating it is unlikely that their employer would provide AI-related training [35]. This hesitancy creates a mismatch between the growing relevance of AI and the readiness of the workforce to engage with it. The European AI Act’s Article 4 on “AI Literacy” could act as a catalyst for change [36]. Starting from February 2, 2025, this directive requires “providers and deployers of AI systems to take measures to ensure, to the best extent possible, a sufficient level of AI literacy among their staff and other persons involved in the operation and use of AI systems.” This legislative push will likely increase the demand for AI-focused training and education in Flanders, creating a significant opportunity for initiatives to close the AI literacy gap and foster a workforce that is ready to thrive in an AI-driven economy.

Overall, the Flemish context was selected because it combines a strong digital infrastructure and high levels of innovation performance with comparatively moderate participation in lifelong learning initiatives. This creates a unique setting in which both the opportunities and the structural barriers to AI adoption in health care can be examined in parallel. Moreover, the Flemish health care system is characterized by a dense network of providers and a diverse workforce, making it particularly suitable for exploring how AI integration may affect professionals at different stages of their careers. Finally, the region’s imminent obligations under the EU AI Act further underscore the urgency of assessing workforce readiness and advancing AI literacy, ensuring that the findings of this study carry both regional relevance and broader European significance.

### Research Methodology

This study investigated the requirements, obstacles, prospects, and needs for AI training among the health care workforce by following an exploratory 3-stage research design (refer to Table 1). Initially, preliminary semistructured interviews (stage 1) were conducted to gather qualitative insights that informed the development of a subsequent survey (stage 2). The survey was designed as an exploratory needs assessment and was not powered for statistical inference. In the third stage, focus groups were used to co-interpret the survey results and extract deeper insights. This multistage approach ensured that the study addressed relevant, context-specific topics and was grounded in real-world perspectives. The study feasibility was assessed through Vlaamse AI Academie (VAIA)’s coordination role in terms of recruitment rates, participant retention, and completion rates at each stage of the study. Acceptability was evaluated, also via VAIA-facilitated feedback processes, based on participants’ ratings and qualitative comments regarding the relevance, clarity, and perceived applicability of the sessions. This study followed COREQ (Consolidated Criteria for Reporting Qualitative Research) and CHERRIES (Checklist for Reporting Results of Internet E-Surveys) guidelines; completed checklists are provided in Multimedia Appendix 1.

**Table 1.** Research design.

Research stage	Stage 1	Stage 2	Stage 3
Research objectives	Gaining insights for the use of AI^a^ in health care	Knowledge, skills, and training needs on AI in health care	Data integration and interpretation
Research tool	Semistructured interviews	Quantitative survey	Three focus groups
Research subjects	Key informants and representatives of medical staff	Medical staff across Flanders	Medical staff across Flanders

a AI: artificial intelligence.

More analytically, in the first stage of the study, the semistructured interview guide was developed through an initial literature review on AI adoption in health care and digital training strategies [3,7]. The guide was subsequently refined in consultation with an expert panel consisting of 3 academic researchers and 2 clinical educators familiar with AI integration and adult learning in clinical settings. Fifteen semistructured interviews were conducted with key informants from the health care sector selected to represent diverse levels of AI expertise (refer to Table 2). These individuals play a crucial role in shaping AI-related training strategies, curriculum development, policy adoption, and institutional decision-making. Their inclusion allowed us to capture insights beyond clinical practice and reflect the broader ecosystem necessary for successful AI integration. The earlier stages of the study (interviews and surveys) were focused predominantly on frontline health care practitioners. The inclusion of nonpractitioners in the focus groups during the final phase allowed us to validate, challenge, and expand the findings with institutional and educational perspectives, leading to more actionable conclusions. At the same time, given the co-interpretive nature of the final research stage, having a diverse group of stakeholders (including those responsible for educational programming, hospital-level innovation, and workforce development) aligned with our participatory research design and enhanced the practical utility of the findings.

**Table 2.** Background of interviewees and artificial intelligence expertise level.

Interviewee	Background	Expertise on AI^a^ (scale 1-5)
Int1	General practitioner	2
Int2	Logopedist	1
Int3	Research nurse	3
Int4	Physiotherapist	4
Int5	Professor of Geriatric Psychiatry, psychotherapist	3
Int6	Professor Dentistry, Periodontologist	4
Int7	Professor of Cardiology and Head of Department	3
Int8	Associate Professor Urology	3
Int9	Cluster Manager Digital Health	3
Int10	Data Sciences Lead Pharmaceutical	4
Int11	Innovation Lead – Hospital	4
Int12	Self-employed home care nurse	1
Int13	Pathologist Hospital	1
Int14	General practitioner	2
Int15	Professor of Internal Medicine and Engineering	5

a AI: artificial intelligence.

A strategic sampling approach combined criterion sampling to target interviewees in pivotal health care roles and maximum variation sampling to ensure a broad spectrum of AI expertise. The incorporation of these methodological stages demonstrated the study’s dedication to obtaining a comprehensive understanding of AI-related learning requirements while encouraging inclusiveness and usefulness in its conclusions. The aim was to explore the use of AI in health care within Flanders and to identify the existing AI training needs of the sector. The interview data were analyzed using thematic analysis [37], supported by NVivo (QSR International) software to systematically identify themes. Following the principles of grounded theory, coding proceeded in three iterative stages: (1) open coding, in which transcripts were examined line-by-line to capture discrete concepts and assign initial codes; (2) axial coding, where relationships among codes were explored and grouped into broader categories by linking subthemes and identifying patterns; and (3) selective coding, which involved integrating these categories around central, overarching themes that addressed the research questions. During the process, a constant comparison approach was applied within and across interviews to refine category boundaries and ensure analytic consistency. NVivo’s memo and annotation functions were used to document coding decisions, emerging theoretical insights, and reflexive notes, creating an auditable record that enhanced the transparency and credibility of the analysis.

In line with qualitative research best practices, data collection for interviews continued until thematic saturation was achieved; that is, the point at which no new codes or themes emerged. Rather than relying on the rather loose criterion of no new data, we assessed theoretical saturation in terms of the completeness and depth of the analysis (Low [38]). This point was reached after the 13th interview, with the final 2 interviews serving to confirm saturation without introducing novel concepts. Reaching this stage indicated that further interviews were unlikely to generate additional analytical insights and thus informed the decision to conclude preliminary data collection at that point.

Building on the insights from the qualitative data, the study proceeded to design a survey aimed at conducting a comprehensive needs assessment [27] across the regional medical workforce. The survey focused on several key areas, including health care professionals’ learning trajectories, their existing knowledge and skills related to AI, the impact of AI on their professional practices, and their specific learning needs regarding AI adoption. Developed using the EU Survey platform, the survey ran from July till the end of December of 2024. Prior to full deployment, the survey instrument underwent pretesting with 5 health care professionals (2 physicians, 1 nurse, 1 health policy advisor, and 1 hospital IT coordinator) to ensure clarity, relevance, and usability of the items. Based on their feedback, minor revisions were made to improve question wording, reduce ambiguity, and streamline the Likert-scale design. Regarding psychometric evaluation, the section on importance-of-topics for AI training consisted of multiple items rated on a 10-point Likert scale. We conducted an internal consistency analysis for this group of items. The resulting Cronbach α was 0.84, indicating good internal reliability of the scale.

The survey was digitally distributed to a wide array of health care organizations across Flanders, including 4 hospitals, 5 health care professional associations, 4 health care–focused Small and Medium-sized Enterprises and enterprise associations, 6 additional Small and Medium-sized Enterprises, 2 university medical schools via their alumni lists, and 1 nongovernmental organization (NGO), namely, VAIA–Flanders AI Academy, with which 5 of the authors are affiliated. Additionally, the survey was shared through personal networks and disseminated via health care channels and conferences in the region. While this approach ensured broad and diverse reach, it also meant that the exact number of individuals who received or engaged with the invitation is unknown, making it impossible to determine a precise denominator or formal response rate. This limitation is a recognized challenge in nonprobability, exploratory research, particularly when targeting hard-to-reach professional populations, such as health care workers. To mitigate institutional bias, survey distribution relied on multiple independent networks beyond VAIA. The study does not aim for statistical generalizability but follows a qualitative, leaning-needs assessment approach where the focus is on depth and diversity of perspectives, rather than representative prevalence estimates.

To mitigate the risk of nonresponse bias, we ensured diversity in occupation, age, and experience among respondents and triangulated survey findings with insights from in-depth interviews and focus groups, enhancing the trustworthiness and validity of our conclusions. Despite extensive efforts, the survey yielded only 139 responses (refer to Table 3) from an estimated total population of 5000 health care professionals, underscoring the challenges of engaging this demographic group in survey-based research [39] (we need to note that the EUsurvey website was offline for 10 days in the first weeks of September, which we consider a very important time frame, since many potential participants returned from their holidays and multiple newsletters were shared). Five of the participants had filled less than 20% of the total questionnaire (more than 80% missing variables) and were excluded from the analysis, resulting in a final number of 134 respondents included in the analysis.

Health care professionals are a notoriously difficult group to engage in survey-based research due to time constraints, workload pressures, and survey fatigue [39]. This is reflected in the survey responses (134 valid), which may appear limited in proportion to the estimated size of the target population (around 5000 professionals). While the challenges for engaging health care professions are well documented, the small sample still included a broad cross-section of health care roles across multiple regions in Flanders. This occupational heterogeneity enhances the analytical utility of the dataset. Most importantly, the survey was not intended as a stand-alone quantitative assessment but as part of a triangulated qualitative methodology that included in-depth interviews and focus groups. These multiple layers provided contextual richness and analytic depth that helped counterbalance the relatively modest survey response rate. Along these lines, the primary aim of the survey was to conduct a needs assessment, rather than to generalize findings statistically. Therefore, this study is positioned within a qualitative research tradition, aligning with existing literature that frames basic survey statistics as supporting exploratory, interpretive aims rather than pursuing broad statistical generalizability [40]. Accordingly, the insights gained were intended to inform subsequent qualitative analysis and cocreation activities, rather than to produce definitive conclusions about prevalence or distribution. This approach aligns with the growing emphasis on qualitative, reflexive research in the field of AI adoption, which has gained momentum in recent years [41]. Nevertheless, we recognize that the survey results should be interpreted as indicative rather than statistically representative of the entire health care workforce. This limitation reflects both the exploratory scope of the study and the methodological challenges of capturing a rapidly evolving field, such as AI in health care. Rather than aiming for generalizability, the survey was designed to generate insights, highlight emerging patterns, and guide the more in-depth qualitative inquiry. This recognition informed our decision to frame the study within an exploratory, qualitative-driven design, where the survey functions as a complementary entry point to contextualize and deepen subsequent analyses.

**Table 3.** Demographics of participants in the survey.

Demographic characteristics		Participants, n (%)
Sex		
Male		47 (35.07)
Female		83 (61.94)
Other and N/A^b^		4 (2.98)
Age (years)		
<30		16 (11.18)
30-40		42 (27.37)
40-50		34 (23.77)
50-60		34 (23.77)
60-70		7 (4.9)
NA		10 (7)
Seniority (years)		
0-3		8 (5.6)
4-10		38 (26.57)
10 more than		88 (58.74)
Occupation		
Physician/doctor		45 (33.58)
Nurse/midwife		41 (30.6)
Manager		9 (6.72)
Dentist		8 (5.97)
Pharmacist		3 (2.24)
Physiotherapist/kinesist		12 (8.95)
Other^a^		16 (11.94)

a Including Podiatrist, Lab supervisor or geneticist, Radiographer, Medical Physicist, Biomedical Informatics (3), Clinical Trial Center employee.

b NA: Not available.

In the final stage, 3 focus groups were conducted in December 2024, involving 39 medical practitioners, nurses, organizers of courses, and lecturers each, to help with an inclusive and interpretive examination of the survey information gathered. The focus groups played a crucial role in analyzing the survey results, allowing stakeholders from the health care sector to validate key findings and translate them into concrete actions (refer to Table S1 in Multimedia Appendix 1 mapping high-level themes to illustrative quotations). Each session lasted approximately 2 hours and was structured around a semistructured facilitation protocol that included (1) presentation of key survey findings, (2) guided discussion on their relevance and interpretation, and (3) open dialog to surface additional perspectives or contextual nuances not captured by the survey. The facilitation was carried out by trained moderators of the core analysis team, with note-takers documenting group dynamics in addition to verbatim audio recordings. The discussions were transcribed in full and subjected to thematic analysis. Coding followed a hybrid approach, with an initial deductive framework based on survey-derived themes and policy priorities, complemented by inductive codes emerging from participants’ reflections and narratives. Coding was conducted independently by at least 2 researchers per transcript, followed by iterative rounds of comparison, discussion, and refinement to enhance transparency and consistency. Intercoder reliability was not treated as a purely statistical exercise but as a qualitative consensus-building process, where discrepancies were discussed until agreement was reached on coding decisions. NVivo software supported the organization of codes, memo-writing, and linking of themes to illustrative quotations. This process ensured that the focus groups were not simply used as a validation step but as interpretive tools that added depth, nuance, and contextual grounding to the survey results. By capturing a broad spectrum of viewpoints and applying systematic procedures for facilitation and analysis, the study strengthened the credibility, rigor, and transferability of its findings.

### Ethical Considerations

This study was conducted in line with ethical standards for research involving human participants. It was carried out under the ethical framework of an ongoing approved project at the School of Political Sciences, Aristotle University of Thessaloniki (ethics approval number 237317/2024). In phase 1, interviewees gave written informed consent, and in phase 2, survey respondents consented via an opt-in form, and no personal identifiers were accessed by the researchers (email handling for prize draws was done solely by a VAIA communication staff member). In phase 3, all focus group participants consented to the use of anonymized ideas and images for scientific purposes. At no point was sensitive personal data collected, and all procedures were designed to ensure participant anonymity and voluntary participation. All consent materials will be made available upon request.

## Results

### Preliminary Insights From Key Informants

In order to inform the design of the following research survey, the preliminary semistructured interviews explored the opportunities and challenges of AI integration in health care. The interviews highlighted a collective emphasis on the need for AI literacy as an overarching concern, as well as the reduction of administrative burdens and ethical considerations arising from AI use as specific content-related issues. Importantly, they highlighted references for learning modalities and the role of accreditation for health care workers. To actively engage health care professionals in relevant training, the most common argument was that AI tools need to provide practical benefits to their daily workflows soon after their training. As one of the interviewees emphasized, “there will be more people interested when it is really practical, when you know how to use it in your daily practice” (Int_1). Similarly, another one pointed out that many professionals have “little time to really delve completely into something new” (Int_2), underscoring the need for tools that integrate seamlessly into existing systems. By implication, online courses were widely preferred, but they were perceived as coming with challenges, such as reduced engagement compared to in-person settings. Although many professionals preferred online learning for its flexibility, a researcher pointed out that specifically, nurses often favor in-person training (Int_3). One way or another, the critical parameter for AI training integration that had to be explored was its contribution to the peoples’ workload.

On this point, the most important potential contribution of AI that emerged from the interviews was related to the overwhelming administrative burden faced by health care professionals, with the use of AI to automate repetitive tasks, such as form completion and report generation, being viewed as a promising solution. Most interviewees consented to the view that health care—and nursing, in particular—could benefit from AI tools to decrease the administrative burden and get more time for the patients. In this context, the head of a cardiology department highlighted that administrative tasks had grown over the years, reversing the time allocation between patient care and paperwork: “If I compare with 20 years ago, my focus was more than 80% on the patient and 20% on the administration. What we do see now is that it is just the opposite” (Int_7). In this context, several key informants shared the sentiment that saving time spent in administration could be the most critical contribution of AI.

The shortage of time was also related to workforce shortages, especially in nursing and psychiatry. Accordingly, AI’s role in assisting with tasks that are increasingly difficult to manage due to workforce shortages “help make the job of a nurse easier to get care where it’s needed” (Int_15). AI’s potential to improve patient care was another focus area. From simplifying patient tracking to offering personalized health information, professionals identified various ways in which AI could benefit patients. For instance, someone stressed that “anything that can ensure that the patient is better informed and gets information tailored to them at the time they are ready” (Int_2) would be valuable. More information does not always come without challenges, as it can exacerbate the tendency to overtest, potentially tipping the balance further in favor of overdiagnosis, as one associate professor of medicine expressed his concerns (Int_8). This perspective highlights the need for careful consideration of the role of AI in health care to avoid unintended consequences. Rather than reducing the administrative burden due to overdiagnosis, AI may ultimately enhance it.

In addition to this, AI’s adoption brings inherent challenges, particularly regarding privacy, data security, and trust in opaque “black box” systems. A doctor underscored this issue, stating, “Everything that we helped validate or set up ourselves is fully trustable, but everything else developed as black boxes outside our environment, I don’t trust at all” (Int_6). This skepticism underscores the importance of transparency and stakeholder involvement in AI development and deployment. Along this line, another interviewee emphasized the critical importance of using AI judiciously. While acknowledging its potential to assist with tasks, such as language translation, he cautioned against overreliance on AI systems, advocating for a balanced approach to ensure its proper application in diverse contexts.

These normative and ethical challenges raise the need for extensive training in order for the integration of AI in health care to come organically. Along these lines, many interviewees agreed on the importance of accredited courses for AI literacy. One of them noted that professionals are drawn to accredited programs because “they get points for the accreditation” (Int_6). In Belgium, many health care professionals must follow continuous professional development (CPD) to maintain their accreditation, which is legally required for some professions. This system ensures they stay up to date and explains why they value training that aligns with their daily practice. However, these courses must directly impact clinical practice to attract interest. As another interviewee observed, “You need something that’s directly influencing the practice before you go to a course” (Int_8). From a research perspective, the challenges and training needs of AI integration in health care must include well-structured training trajectories grounded in extensive, multidisciplinary research. This is where the current research survey plays a pivotal role, offering wide insights across diverse professional groups and health care settings.

### Mapping AI Training Trajectories

Informed by the preliminary interviews with key informants, the second stage of this research used a survey to evaluate the learning needs and interests of health care professionals in Flanders regarding AI literacy in health care. The survey garnered responses from a diverse group, including doctors, nurses, and health care managers, revealing both a strong interest in AI literacy and significant gaps in existing knowledge and training.

**Figure 1.**
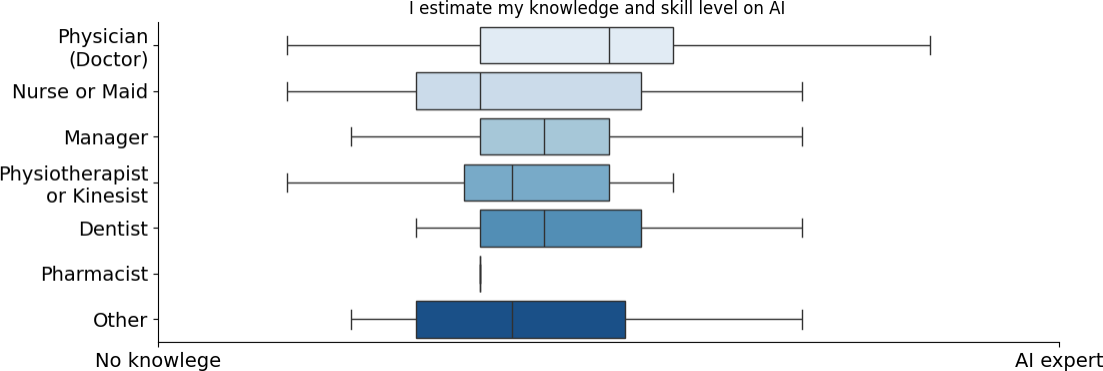
Self-estimation of artificial intelligence knowledge based on occupation. AI: artificial intelligence.

**Figure 2.**
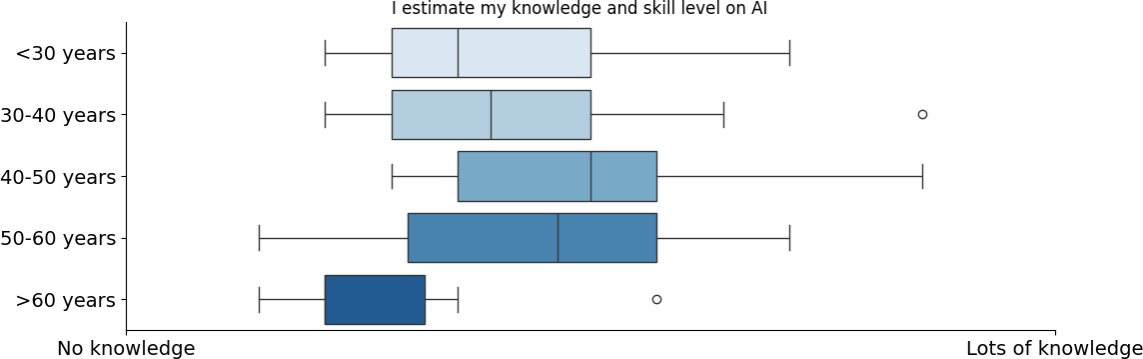
Self-estimation of artificial intelligence knowledge based on age. AI: artificial intelligence.

**Figure 3.**
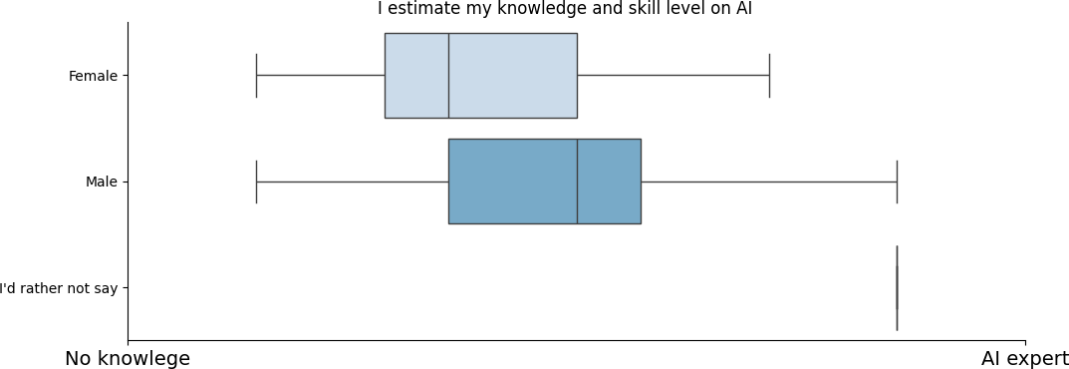
Self-estimation of artificial intelligence knowledge based on sex. AI: artificial intelligence.

More analytically, a significant survey indication was that AI knowledge levels vary across different professions. Managers, physicians, and dentists reported higher AI expertise while nurses, physiotherapists, and pharmacists showed lower familiarity (refer to Figure 1). This indicated that AI exposure is not uniform across medical fields, with some roles integrating AI more actively than others. Age played a crucial role in these variations, as expected. Midsenior professionals (40‐50 years old) reported the highest levels of AI knowledge, whereas older professionals (>60 years old) had the least, with some indicating no AI expertise at all (refer to Figure 2). Responses from the age group of 50‐60 years were mixed, suggesting varying degrees of AI exposure within this demographic. The age group of 50‐60 years and the physicians exhibited the highest SD in their replies. Contrary to what was initially expected, we can see that younger ages (<40 years) are not yet as familiar with AI, indicating the fact that AI is not yet part of the main curricula of the universities in the health care faculties but a knowledge obtained by individuals in a later stage (either by postgraduate programs or by extra training courses). The only question where we observed a sex-based difference in responses was related to self-estimated AI knowledge (Figure 3). Male respondents generally reported higher self-estimates than female respondents. However, when analyzing responses within the same occupation, the differences disappeared. The overall sex disparity arises because our sample includes more female nurses (who tend to report lower AI knowledge self-estimations) and more male physicians, who report higher self-estimations. Therefore, the apparent sex difference shown in Figure 3 primarily reflects an occupational disparity rather than a true sex-based variation.

**Figure 4.**
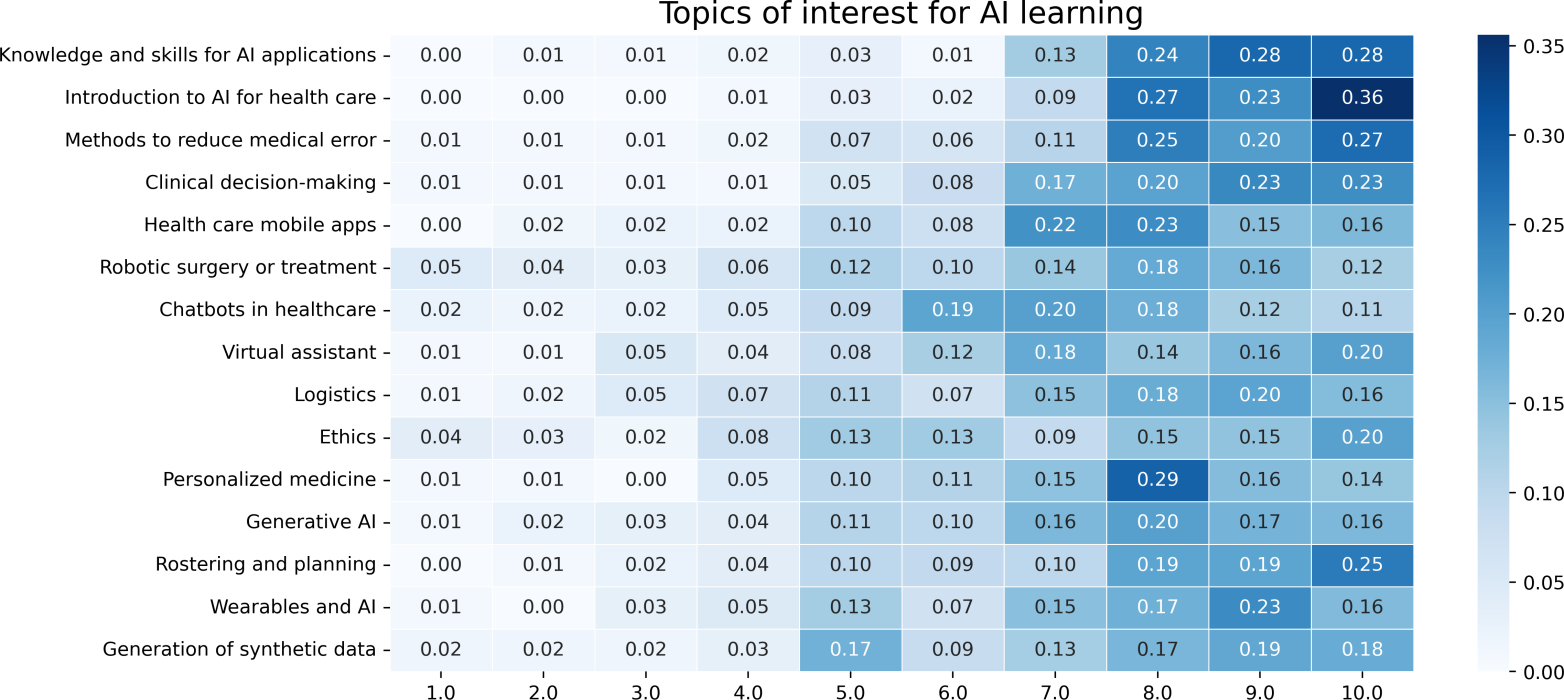
Topics of interest for artificial intelligence learning (Likert scale 1-10). AI: artificial intelligence.

Concerning the type of knowledge that the respondents would like to acquire for AI, most respondents prioritized “gaining basic AI knowledge” (74.62%, 100/134), learning to “be able to facilitate the use of AI in my field (design a study, management, knowledge of ethics, etc)-no coding” (70.29%, 94/134), and “efficiently use predeveloped AI tools” (66.42%, 89/134). Doctors and dentists were the 2 groups that were more interested (75.37%, 101/134 and 72.39%, 97/134, respectively) in the facilitation of AI use in their field rather than the efficient use of predeveloped AI tools. Related to the exact topic of an AI course that they would like to follow, a significant majority of respondents expressed strong interest in an introductory AI course tailored to health care (85.07%, 114/34), with nearly as many (80.6%, 108/134) eager to acquire practical AI skills relevant to their field (refer to Figure 4). Learning priorities varied by age, with younger professionals (<30 years old) actively seeking AI education, especially in decision-making and generative AI, while older professionals (>50 years old) showed less interest in learning AI but acknowledged its role in reducing errors and improving patient confidence. The age group of 30‐50 years balanced curiosity with concerns about practical implementation. These findings suggest AI education should be tailored for younger professionals and practical implementation strategies for older ones. The survey further highlighted a strong preference for immediately applicable AI tools over advanced technical expertise.

No significant difference was noted between respondents of different genders, while again a variability of the choices based on the occupation has been identified. The 2 most interesting topics for almost all the occupations are the first two of Figure 4, “Knowledge and skills for AI applications” and “Introduction to AI for Healthcare.” For nurses, though, “Rostering and planning” appears as the second most interesting topic (after the “Introduction to AI for Healthcare”), a topic that is much lower in the selection of the other occupations. “Personalized medicine” appears to be the third option for dentists, others, and physicians (doctors), while “Methods to reduce medical error” appears more important for managers, nurses, and pharmacists. Another interesting finding is the fact that, contrary to previous studies [27], the topic of “Ethics” is low in the interest of the respondents. The least interesting of the topics among almost all the groups (except for dentists, for whom it was penultimate) was the “Generation of synthetic data.”

Different occupations exhibited distinct patterns in their training preferences and frequency. Dentists and clinicians were the most engaged in continuous learning, with the majority attending training sessions at least once per month. In contrast, professionals in other occupations typically participated in training once every 3 months or less. When selecting a course provider or topic, dentists, clinicians, and other health care professionals primarily trusted specialists in their respective domains, followed by their training institutions (universities or technical schools). However, physiotherapists and nurses prioritized recommendations from their employers over their training institutions. Managers stand out as the only group that places the highest trust in their employees’ suggestions regarding the choice of courses, rather than relying on peers or experts in their field. Across all groups, social media and government recommendations play only a small role in influencing training choices.

**Figure 5.**
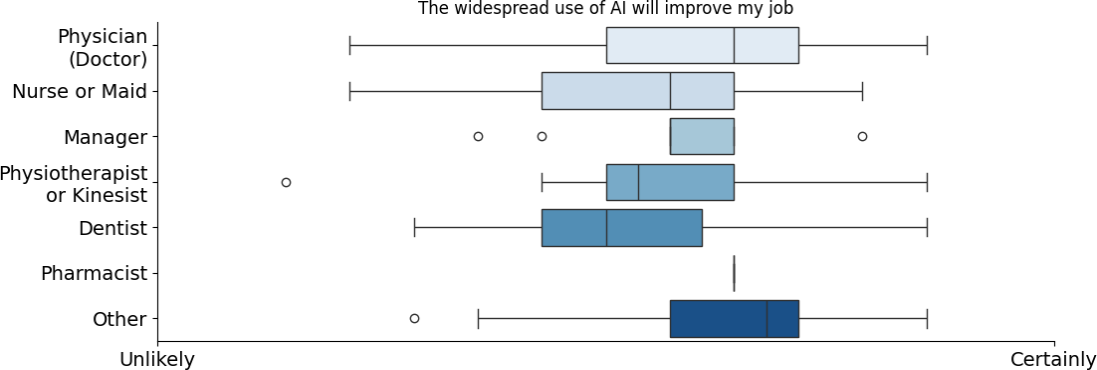
Opinion about the improvement that artificial intelligence will bring into their job by occupation. AI: artificial intelligence.

**Figure 6.**
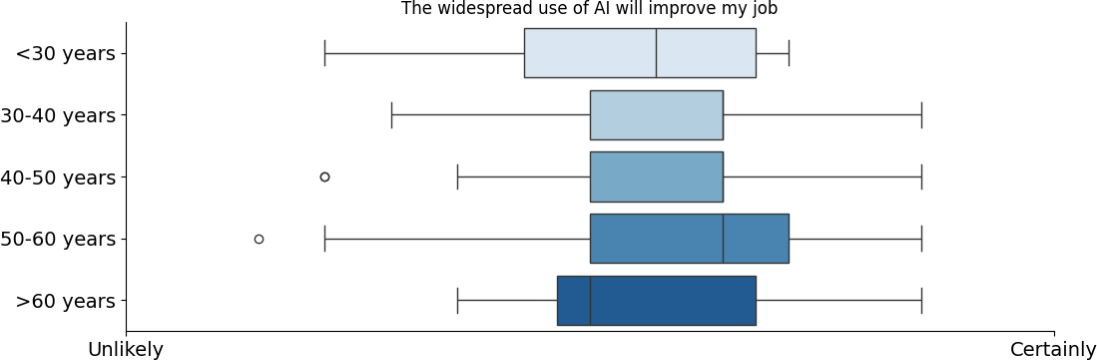
Opinion about the improvement that artificial intelligence will bring into their job by age. AI: artificial intelligence.

In parallel, perceptions of AI’s impact on improving health care jobs varied by profession. Physicians and nurses were the most optimistic while dentists and physiotherapists were more skeptical (refer to Figure 5). Managers and pharmacists held moderate to positive views. Similarly, younger professionals (<30 years old) strongly believed AI would enhance their work, whereas older professionals (>50 years old) were more cautious (refer to Figure 6). A notably greater SD was also observed among doctors and individuals aged 50‐60 years, which may reflect the diversity of clinical specialties within these groups (such as cardiology, neurology, and others) that influence their experiences and attitudes toward AI integration. This heterogeneity suggests that the field of practice could be a key moderating factor underlying the observed variability [42]. Regarding AI’s future in health care, professionals identified its greatest contributions as increasing patient confidence, reducing diagnostic bias, enhancing decision support, and improving access to health care information (refer to Figure 6).

**Figure 7.**
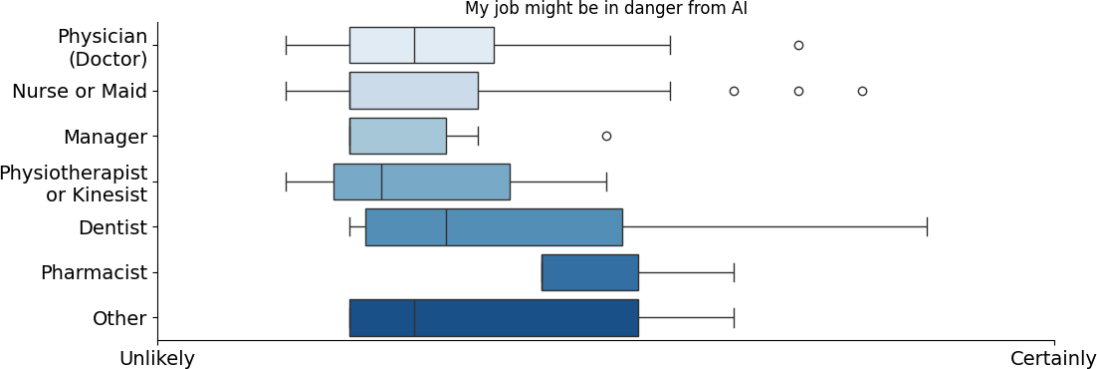
Opinion of the participants by occupation about the danger that artificial intelligence might bring to their job. AI: artificial intelligence.

**Figure 8.**
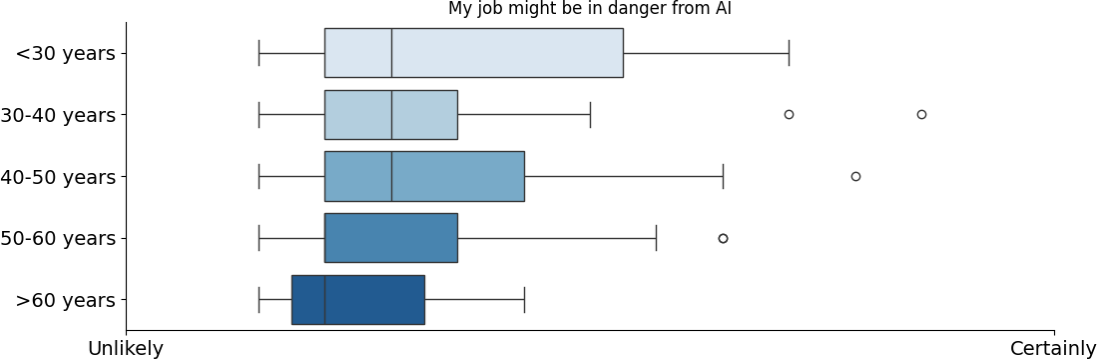
Opinion of the participants by age about the danger that artificial intelligence might bring to their job. AI: artificial intelligence.

An interesting finding of the survey was how health care professionals perceive the risk AI poses to their work. Again, AI perception in health care varied significantly by profession, age, and potentially gender. Dentists and pharmacists felt the highest risk of AI replacing their jobs, likely due to automation in diagnostics and prescriptions. Physicians and nurses were more confident, with a broader distribution of responses (refer to Figure 7). Age also played a role, as younger professionals (<30 years old) felt more at risk, whereas older professionals (>50 years old) were less concerned, although neither was significantly concerned (refer to Figure 8). This generational difference suggests that younger professionals, entering a rapidly evolving workforce, may feel more vulnerable to technological changes, whereas older professionals, with more established careers, perceive AI as less of a direct threat.

Younger professionals were more concerned about job displacement, likely because they recognized AI’s growing role in health care and wanted to stay competitive. This contrast reflects a mix of ambition and modest anxiety among younger professionals versus confidence and stability among older ones. As mentioned by Webb [43], the impact of AI on employment may manifest more through reduced entry into occupations—fewer young people starting these jobs—rather than through increased exits by older workers, leading to a disproportionate effect on younger workers, and this “entry margin” effect might be reflected in the answers of the participants. If you consider the answers based on the occupation, we can note that pharmacists and dentists are the 2 groups that were slightly more concerned about their jobs and believed that AI will improve their jobs less (refer to Figures5  6, respectively).

**Figure 9.**
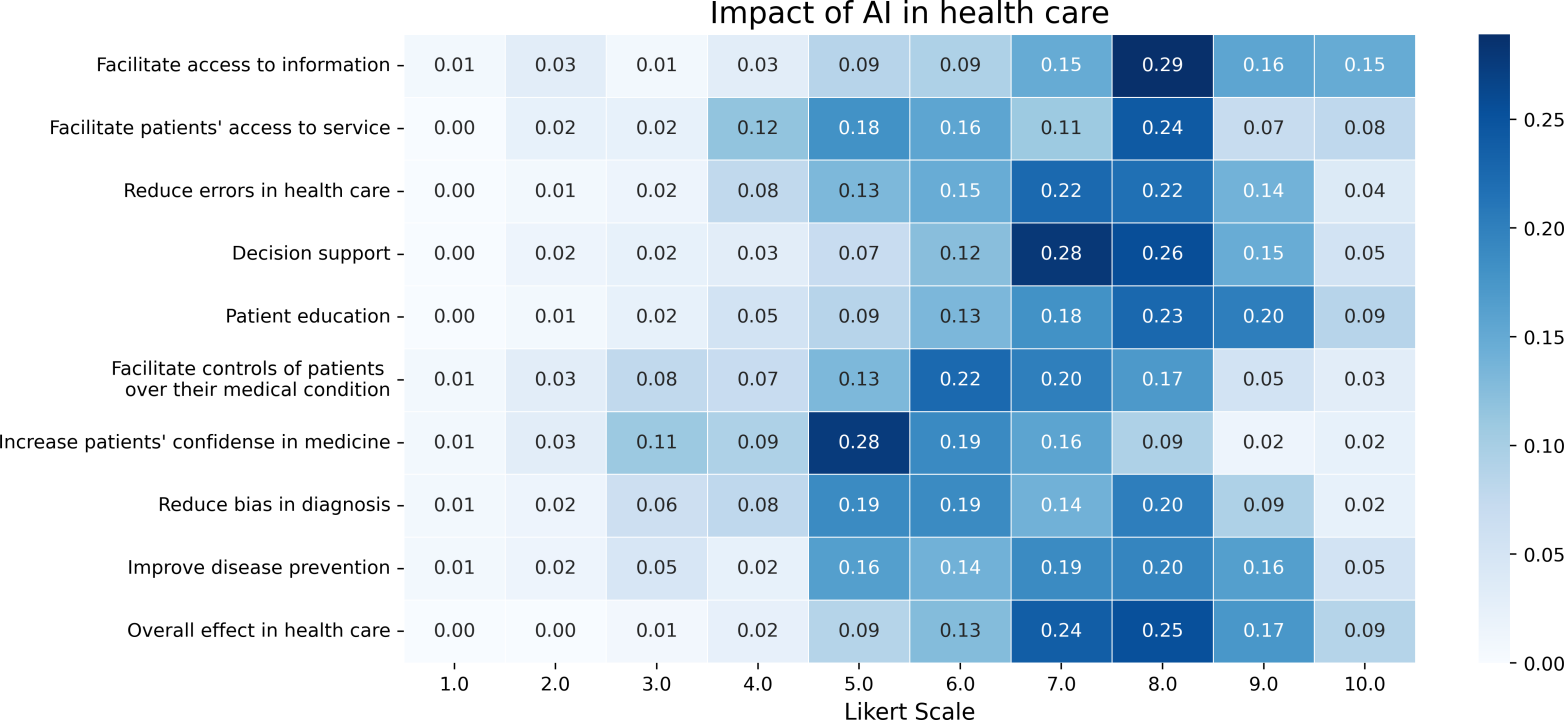
Impact of artificial intelligence in health care. AI: artificial intelligence.

The responses regarding the area where AI will have the greatest impact were quite diverse (refer to Figure 9). Participants generally agreed that AI would influence almost all the mentioned fields (access to information, patients’ access to service, reduction of errors in health care, patients’ education, control of patients over their medical conditions, reduction of bias in diagnosis, improvement of disease prevention, and overall effect in health care). The only exception (or at least the area with the fewest responses based on the Likert score) was “increasing patients’ confidence in medicine.” The most significant areas of impact, though closely followed by others, were “facilitating access to information,” “overall healthcare,” and “decision support systems.” Notably, no significant differences were observed across various demographic categories, including gender, age, occupation, or experience level.

### Co-interpreting AI in Flanders’ Health Care

The latest stage of this study focused on interpreting data through a collaborative framework of focus groups. A total of 39 participants took part in 3 focus groups, representing key stakeholders in health care and education, including lecturers from various disciplines (excluding practitioners), education and innovation hospital managers, representatives of nursing and radiology associations, and members of health care communities. To ensure efficient discussion and facilitation, the groups were structured as follows: 14 participants in the first group, 12 in the second, and 13 in the third. The focus groups were conducted in late December 2024, following a presentation of the initial survey results by the last author. Groups 1 and 3 were facilitated by the first author, while Group 2 was led by the last author. Groups 1 and 2 took place simultaneously following the “Split Groups Discussion” methodology [44]. Each group provided distinct perspectives on the integration of AI in health care, yet several overarching concerns emerged, particularly regarding generational differences in attitudes toward AI, trust in technology, and the evolving role of health care professionals. The demographic information of the participants in the round tables discussion can be found in Table 4.

**Table 4.** Demographics of participants in round tables discussions.

Demographic characteristics	Participants, n (%)	
Sex		
Male		12 (30.76)
Female		27 (69.24)
Age (years)		
<30		1 (2.56)
30-40		9 (23.08)
40-50		12 (30.77)
50-60		4 (10.26)
NA		13 (33.33)
Occupation		
Manager		12 (30.77)
Lecturer		9 (23.08)
Nurses/midwives		4 (10.26)
Physiotherapist/kinesist		2 (5.13)
Course provider		9 (23.08)
Other		3 (7.69)

Each focus group provided unique perspectives on AI integration in health care, confirming several key survey insights while adding deeper practical and ethical dimensions to the discussion. Echoing survey responses, all 3 groups emphasized AI’s role in reducing administrative burden, with particular interest in AI-driven solutions for documentation, scheduling, and workflow optimization. Discussions in the focus groups revealed a range of perspectives on AI in health care, with a particular emphasis on differences between professional groups, challenges in training, regulatory barriers, and disparities in available resources.

One of the central themes raised in the discussions was the difference between nurses and doctors in their access to and engagement with AI training. Participants noted that AI tools are primarily designed for doctors, while significantly fewer applications exist for nurses. This was seen as a major limitation, making it difficult for nurses to integrate AI into their daily routines. Additionally, nurses and doctors follow training at different times. Nurses prefer courses during working hours and often expect compensation, whereas doctors tend to attend evening sessions. Some participants expressed frustration that funding for AI training is more readily available for doctors and research-oriented professionals while nurses have far fewer opportunities.

A recurring concern was the difficulty of developing hands-on AI courses. Many participants pointed out that the most relevant knowledge on AI implementation resides within private companies, but these companies are not interested in creating educational programs; rather, their focus is on selling AI products. This dynamic created a gap in practical training, as health care professionals needed guidance on AI applications but did not trust company-led training initiatives, rather than courses coming from universities. A few participants mentioned that radiologists were an exception, as companies do provide AI training in collaboration with the Radiology Society. However, in most other fields, there was a clear demand for cocreated courses where universities and companies work together to ensure both credibility and relevance.

Another major topic discussed was the regulatory complexity surrounding AI in hospitals. Participants frequently emphasized that many AI tools were not allowed to be used in clinical practice, creating uncertainty about what is permitted. There was a strong sentiment that hospitals need clearer guidelines and policies to help professionals navigate AI implementation. Some participants felt that the lack of clarity leads to hesitation, making AI adoption slower than it could be.

Time constraints for training were another issue raised in the discussions. Doctors, especially those in private practices, had more flexibility in choosing when to follow AI courses, while nurses required training during work hours, which limited their opportunities. There was agreement that hospital management plays a crucial role in ensuring all health care professionals, not just doctors, have access to AI education. Several participants pointed out that doctors, who often earn more by seeing more patients, may have a financial incentive to adopt AI tools that improve efficiency, while nurses, who work on fixed salaries, focus more on how AI can support patient care and workflow improvements (supported also by the survey and their interest in courses related to rostering and planning). Participants also discussed the general lack of resources for AI education. There was widespread agreement that funding constraints made it difficult for hospitals and universities to develop robust training programs, especially for lifelong learning. Some attendees noted that universities tend to focus on AI education for students rather than professionals, as resources are limited, and institutional priorities are not always aligned with the needs of the workforce. A broader issue raised in the discussions was the lack of ready-to-use AI tools in clinical practice. Many participants emphasized that health care professionals need AI solutions that can be integrated immediately into their workflows, yet such tools are not widely available. Some expressed frustration that AI was often presented as a future technology rather than something that can help them today. Others pointed out that existing AI training often focuses on theoretical knowledge rather than practical applications.

A particularly interesting discussion arose around ethical considerations. While ethics courses are often integrated into broader AI programs, the focus groups revealed a clear generational divide in how participants approached AI ethics. Some participants pointed out that younger professionals, particularly students, tend to prioritize ethical concerns (as has also been pointed out by previous studies conducted with students [27]), whereas professionals in the workforce are more focused on AI as a functional tool. A common argument was that health care workers simply do not have the time to engage deeply with ethical discussions in their daily routines and believe that ethical concerns should primarily be handled by AI developers. However, there were diverging views within the groups. One participant argued that financial incentives drive much of the AI interest in health care, particularly among doctors who earn more with increased patient volume. In contrast, another participant from a postgraduate palliative care clinic shared that ethics was a major focus in their discussions, suggesting that ethical concerns become more prominent in fields dealing with sensitive end-of-life care. This small disagreement highlighted that while some professionals may deprioritize ethics, others see it as essential depending on the context in which AI is applied.

Across all 3 groups, discussions highlighted a complex and evolving landscape for AI adoption in health care. In Table 5, the key cross-cutting themes (ie, administrative burden, ethics, training logistics, trust, and generational and occupational differences) can be viewed. It illustrates how each was supported by data from interviews, surveys, and focus groups. While there was general recognition of AI’s potential, participants made it clear that their trust, learning needs, and expectations vary significantly based on their profession, work environment, and access to resources. Addressing these concerns will require targeted training strategies, clearer regulations, and better collaboration between academia, industry, and health care institutions.

**Table 5.** Joint display of the cross-cutting themes in the 3 different phases of the study (ie, inverviews, surveys, and focus groups).

Theme	Interview insights	Survey findings	Focus group insights
Administrative burden	Viewed AI^a^ as a potential tool to reduce time spent on documentation and form-filling	Identified as a high-impact area for AI use, especially by nurses and midcareer professionals	Strong consensus that administrative relief is AI’s most practical benefit in the short term
Ethical concerns	Raised, but often secondary to practical concerns. Some distrust in “black box” systems	Ethics ranked low in topic interest, especially low among clinicians	Disagreement between participants Opinion for decreased importance of ethics once you graduate and start working, whereas palliative care workers emphasized ethical sensitivity
Training logistics	Online is preferred for flexibility, but in-person is better for engagement Nurses need compensated training Call for accredited courses	Minor variability among different ages and occupations Everyone wants accredited courses	Gaps in training availability were noted, especially for nurses Call for accredited, hands-on programs
Trust and transparency	AI developed in-house is seen as more trustworthy than external, opaque systems	Trust in AI is lower among older and nonclinical respondents Survey shows moderate optimism but limited usage	Professionals want clearer regulatory guidelines Co-development seen as a way to build trust
Occupational differences	Nurses need AI for workflow Doctors focus on diagnostics;	Clear role-based differences in learning needs and perceived usefulness of AI	Frustration over unequal access to training funding Nurses feel underserved
Generational differences	Not observed	<30 years of age group most interested in learning AI >50 years of age group least engaged but acknowledge AI benefits	Generational divide on ethics, tool adoption, and openness to innovation

a AI: artificial intelligence.

## Discussion

### Principal Findings

The current multifaceted, qualitative study provided a nuanced view of both opportunities and challenges in integrating AI into health care. The study showed that while the vast majority of respondents recognized AI’s potential to improve efficiency, it also revealed a complex relationship between age, occupation, resource availability, and attitudes toward AI in health care. Younger professionals, nonphysicians were generally more concerned about AI potentially replacing jobs, yet they remained more positive about using AI in their work. This dual perspective is likely linked to their limited access to resources, such as institutional support and opportunities for professional development, and the fact that AI is not yet implemented in the bachelor programs of any medical (or para-medical) university or school in Flanders. Early-career professionals, facing restricted resources and administrative pressures (especially nonphysicians), may see AI as a valuable tool to alleviate workload and enhance their capabilities, making them more eager to integrate AI into their practice despite concerns about job security. In contrast, older professionals (>60 years old) appear less concerned about AI replacing jobs but are more skeptical about actively using AI. This skepticism may stem from their well-established positions and greater access to resources, including institutional support, funding, and established networks, which reduce their perceived need for AI. Having navigated the early stages of their careers, they may feel less urgency to adopt new technologies, especially when their roles are more secure and supported. Essentially, their resource-rich environment provides them with stability, reducing their perceived risk from AI but also limiting their motivation to engage deeply with new technology. These observed generational and occupational divides directly inform our proposed multitiered strategy. Younger, resource-constrained professionals call for subsidized and practice-oriented training schemes while older, resource-secure professionals highlight the need for embedding AI within existing CPD frameworks rather than new standalone programs. By explicitly mapping these divides to differentiated policy interventions, we ensure that recommendations are grounded in lived workforce realities.

Occupations in health care show varying levels of exposure to AI. Clinicians, such as radiologists and lab technicians, face high exposure due to AI’s growing capacity for data analysis and pattern recognition. In contrast, roles like nurses and care managers (where social intelligence, adaptability, and hands-on judgment are essential) are less exposed and more resilient to automation. This does not mean clinicians are at risk of replacement, but rather that their tasks are shifting. To stay relevant, they will need to adapt by integrating AI into their workflow, focusing on what AI cannot do, such as contextual reasoning, communication, and ethical decision-making. Meanwhile, roles with strong interpersonal and organizational components may see their value reinforced, provided they also adapt to AI-enhanced systems [45]. While AI adoption might hold the potential to promote fairer resource allocation, offering individuals in socioeconomic constraints enhanced access to knowledge, skills, and opportunities. The very same groups might also be more vulnerable to job displacement and precarity, as AI-driven transformations may exacerbate existing inequalities rather than alleviate them. The uneven trust and adoption patterns we observed across age and occupational groups illustrate how digital divides are reproduced within the workforce itself, making equity considerations not only a systemic policy concern but also an empirically grounded outcome of our study. This alignment between our data and broader digital equity debates underscores why workforce training cannot be designed as a “one-size-fits-all” model but must instead be differentiated by age, occupation, and institutional support.

These findings are embedded within a broader discussion on digital health equity. The digitalization of health care carries the risk of deepening existing health and social inequities if not accompanied by deliberate, equity-oriented policy frameworks. As digital tools increasingly mediate access to services and reshape clinical workflows, the potential for exclusion becomes particularly acute among already marginalized groups. This has been observed in multiple contexts, including the Netherlands, where digital care policies are framed as progressive and universally beneficial, yet structural risks, such as disparities in digital literacy, socioeconomic access, and institutional support, receive limited attention [46]. Such gaps reflect what has been described as a policy “blind spot” [46], the tendency to depoliticize digital care and detach equity considerations from discussions of infrastructure and governance. In contrast, this study’s results resonate with research highlighting digital literacy training as a core enabler of equitable digital health [47]. This perspective treats digital health, including data and AI applications, not as a neutral technological progression but as a socially embedded transformation shaped by uneven resource distributions, institutional priorities, and workforce dynamics. For example, Danish digital health policy has struggled to reconcile legal commitments to equality with the realities of digital stratification [48]. Similarly, the Flemish context illustrates how even innovation-leading regions may fall short in translating ambition into inclusive practice. In this light, digital health equity must be approached as a governance concern, rather than merely a design feature. In our case, the absence of these enabling conditions—particularly tailored training and institutional recognition of diverse learning needs—contributed to uneven levels of trust in AI and unequal adoption patterns. Nurses emphasized rostering and workflow integration while physicians and dentists were more focused on clinical facilitation. However, few training initiatives accounted for these differentiated needs. This occupational stratification reflects broader patterns of digital exclusion described in the literature, where standardized solutions fail to address context-specific barriers [49]. By explicitly linking these findings to policy debates, such as the EU AI Act, our study shows how occupational and generational inequities in AI adoption mirror broader structural risks, reinforcing the urgency of equity-driven governance frameworks.

Along these lines, we propose a multitiered strategy aligned with key stakeholder roles. First, health care institutions (eg, hospitals and professional associations) should embed mandatory data and AI literacy modules into existing CPD frameworks, ensuring these are accredited, occupation-specific, and available in both online and in-person formats. Training content should prioritize practical integration (eg, rostering tools, triage systems, and clinical decision support) and include embedded ethical scenarios rather than separate ethics modules. Second, regional health authorities and ministries should establish targeted funding schemes that enable subsidized training for underresourced staff groups (particularly nurses) and early-career professionals. These schemes should recognize the structural disadvantage some groups face in accessing noncompensated learning time. Also, providing equal chances for AI literacy for all health care professionals will improve the qualitative adoption of AI systems. Parallel to this, employers should be incentivized (eg, via tax credits or public grants) to allocate protected time for (data and AI) training during work hours.

Third, academic institutions and universities should codevelop interdisciplinary AI education tracks in collaboration with clinical partners and technology developers. These tracks should offer microcredentialing (as came out from our study since accreditation was a request) pathways and include hands-on exposure to real-world AI tools, ideally integrated into existing clinical education curricula to reduce learning redundancy. While concerns regarding the ethical implications of AI in health care were frequently raised, ranging from bias in AI algorithms to patient data privacy, there was surprisingly little interest in dedicated training programs focused solely on AI ethics. This discrepancy suggests that while ethics remains a key concern, it is not seen as an immediate priority for skill development among health care professionals, who are more focused on practical AI applications and workflow integration. Given this reality, embedding ethical considerations within broader AI training, rather than presenting them as standalone courses, may be a more effective strategy. By integrating discussions on bias, transparency, and accountability into technical and clinical AI education, ethics can become a natural part of AI adoption rather than an isolated, optional subject. This approach ensures that health care professionals develop a nuanced understanding of AI’s ethical challenges while remaining engaged in training that aligns with their immediate professional needs. This generational and occupational divergence helps explain why ethics was acknowledged as important yet deprioritized in practice. For younger professionals facing immediate workload pressures, applied utility outweighed abstract ethical debates, whereas older, more established professionals expressed skepticism rooted in systemic trust and governance issues. Importantly, this divergence supports our recommendation to embed ethics within practice-oriented modules, ensuring that ethical reasoning develops in parallel with technical competence rather than in isolation.

Fourth, technology providers must be engaged as co-designers of training, but under strict ethical and regulatory oversight. Partnerships with trusted educational bodies (eg, medical colleges and nursing federations) can help mitigate bias and ensure neutrality while also fostering greater clinician trust in AI tools. Finally, to overcome the fragmentation in training provision and ensure inclusivity, we recommend establishing a regional AI training coordination body, led by health ministries or national digital health agencies, which would curate, accredit, and evaluate AI-related training offers across providers, occupations, and settings. By operationalizing these stakeholder-specific roles, training efforts can be scaled in a way that reflects real-world needs, acknowledges occupational and generational divides, and upholds digital equity as a core principle of health system innovation.

By placing our findings within the broader discourse on digital health equity, we echo recent calls for a multilevel approach to digital transformation—one that embeds structural awareness into both policy development and training implementation. Without such a shift, digital care risks reinforcing the very disparities it promises to address, particularly as regions prepare for the rollout of EU legislation, such as the AI Act, which will impose new obligations on providers and deployers to ensure AI literacy. Given the uneven starting conditions, regional health authorities and ministries should act first by establishing funding schemes and coordination bodies to reduce disparities in access to AI training. This creates the enabling environment in which health care institutions and employers can embed structured training into CPD frameworks. Building on this foundation, universities and technology providers can co-develop and scale interdisciplinary, practice-oriented modules under clear ethical oversight. Our study thus contributes not only to the literature on AI integration in health care but also to the wider debate on how to build equitable digital futures in health systems already marked by occupational, generational, and structural divides.

Concerning limitations of our study, we reckon that the findings are exploratory and based on a nonprobability sample, which means they should be interpreted as indicative rather than representative. The focus on the Flemish context also limits the transferability of results to other health care systems. Moreover, the rapidly evolving nature of AI in health care means that attitudes and readiness may shift over time. At the same time, participation may have been skewed toward individuals with strong opinions, either highly enthusiastic about AI or skeptical of its role in health care, potentially underrepresenting the broader, more neutral majority. Given an estimated distribution, we recognize that our sample overrepresents physicians, nurses, and dentists relative to their actual proportions in the health care system and underrepresents allied health professionals. Comprehensive and up-to-date statistics detailing the full distribution of health care professionals by occupation specifically for the Flemish region are not publicly available in an aggregated format. Nurses (~45%‐50%), physicians (~15%‐20%), dentists (~2%‐3%), and allied health professionals, including physiotherapists, occupational therapists, imaging technologists, laboratory staff, etc (~25%‐30%) [50-52]. Furthermore, the relatively limited number of respondents constrains the ability to conduct robust analyses of intersectional factors, such as examining smaller subgroups defined by combinations of profession, age, or other demographic variables. This limitation reduces the statistical power and granularity needed to uncover nuanced trends within these intersections. This imbalance likely reflects a response bias, as physicians, nurses, and dentists are more frequently targeted in institutional mailing lists and professional networks and may also be more accustomed to participating in research or policy consultations. In contrast, allied health professionals often have less visibility in centralized databases and may face greater workloads or institutional barriers that limit their participation in voluntary research studies and probably also training. Future research should therefore build on these insights through larger-scale and comparative studies, as well as longitudinal approaches that capture changing dynamics.

### Conclusion

Crucially, the findings suggest that a one-size-fits-all approach to AI training is inadequate. Instead, future learning trajectories must be stratified by occupation, age, and available resources, and they should prioritize hands-on applications over theoretical content. Embedding ethical considerations into practical modules—rather than isolating them—may also bridge the current disconnect between ethical awareness and perceived learning relevance. As Flanders prepares for the implementation of the EU AI Act, this research highlights a timely opportunity to cocreate inclusive, accredited, and accessible AI training programs that reflect the lived realities of health care professionals. Doing so will be essential not only for scaling AI integration locally but also for informing broader European and global efforts to build an equitable, AI-ready health care workforce.

## Supplementary material

10.2196/76709 Multimedia Appendix 1 Mapping of focus group themes to illustrative quotation.

10.2196/76709 Checklist 1 COREQ checklist

10.2196/76709 Checklist 2 CHERRIES checklist

## Abbreviations

AI: artificial intelligence
CHERRIES: Checklist for Reporting Results of Internet E-Surveys
COREQ: Consolidated Criteria for Reporting Qualitative Research
CPD: continuous professional development
EU: European Union
EU AI: European Union Artificial Intelligence
NGO: nongovernmental organization
NUTS1: Nomenclature des Unités Territoriales Statistiques
VAIA: Vlaamse AI Academie
