# Supplementary material for: Bridging the AI-Literacy Gap in Health Care: Qualitative Analysis of the Flanders Case Study
Source: J Med Internet Res. 2025 Dec 8;27:e76709. doi: 10.2196/76709 (PMC12685233; doi:10.2196/76709)
Supplement: Multimedia Appendix 1 [file jmir-v27-e76709-s001.docx]

# Multimedia Appendix 1

**Table S1:** Mapping of Focus Group Themes to Illustrative Quotation

| **Theme** | **Representative Quote** | **Focus Group / Participant** |
| --- | --- | --- |
| Practical integration of AI | “You need something that's directly influencing the practice before you go to a course.” | FG2 – Senior physician |
| Inequities in training access | “Nurses want training during work hours, but we often don’t get the same funding or flexibility as doctors.” | FG1 – Nurse association representative |
| Trust in AI tools | “If we didn’t co-develop it, I don’t trust it. These black-box systems worry me.” | FG3 – Academic clinician |
| Regulatory and legal uncertainty | “We don’t even know what’s allowed yet. Hospitals are waiting for clear policies before using these tools.” | FG1 – Innovation manager |
| Ethical concerns and generational divide | “Students are very concerned about AI ethics. In real-world practice, we just need something that works for patients.” | FG2 – Clinical educator |
| Resource limitations in AI rollout | “Universities are not equipped to train the workforce—resources are going to pre-service education, not to professionals.” | FG3 – Medical school lecturer |
| Differences in motivation by profession | “Doctors are looking for time-saving tools; nurses are asking how AI can support patient care more directly.” | FG1 – Hospital training coordinator |
