# Supplementary material for: Bridging the AI-Literacy Gap in Health Care: Qualitative Analysis of the Flanders Case Study
Source: J Med Internet Res. 2025 Dec 8;27:e76709. doi: 10.2196/76709 (PMC12685233; doi:10.2196/76709)
Supplement: Checklist 1 [file jmir-v27-e76709-s002.pdf]

COREQ Checklist (Consolidated Criteria for Reporting Qualitative Research)

Item	Domain	Description	COREQ Question	How addressed in the study
1	Research team	Interviewer/facilitator	Which author/s conducted the interview or focus group?	Core analysis team; trained moderators led focus groups
2	Research team	Credentials	What were the researcher's credentials? (e.g., PhD, MD)	Academic researchers, professors, clinical educators
3	Research team	Occupation	What was their occupation at the time of the study?	Professors, clinicians, researchers, innovation leads (Table 2)
4	Research team	Gender	Was the researcher male or female?	Not explicitly reported
5	Research team	Experience and training	What experience or training did the researcher have?	Expert panel refinement; moderators trained in facilitation
6	Relationship	Relationship established	Was a relationship established prior to study commencement?	No prior relationship mentioned
7	Relationship	Participant knowledge of interviewer	What did the participants know about the researcher? (e.g., personal goals, reasons for conducting the research)	Not detailed
8	Relationship	Interviewer characteristics	What characteristics were reported about the interviewer/facilitator? (e.g., bias, assumptions, reasons and interests in the research topic)	Reflexivity addressed via NVivo memos and annotations

9	Study design	Methodological orientation	What methodological orientation was stated to underpin the study? (e.g., grounded theory, discourse analysis, ethnography, phenomenology, content analysis)	Thematic analysis, grounded theory principles
10	Study design	Sampling	How were participants selected? (e.g., purposive, convenience, consecutive, snowball)	Criterion + maximum variation sampling for interviews; purposive for focus groups
11	Study design	Method of approach	How were participants approached? (e.g., face-to-face, telephone, mail, email)	Invitations via networks, institutions, VAIA
12	Study design	Sample size	How many participants were in the study?	15 interviews, 134 valid survey respondents, 39 focus group participants
13	Study design	Non-participation	How many people refused to participate or dropped out? Reasons?	Not reported
14	Setting	Setting of data collection	Where was the data collected? (e.g., home, clinic, workplace)	Healthcare/academic settings; focus groups in organized sessions
15	Setting	Presence of non-participants	Was anyone else present besides the participants and researchers?	Note-takers present in focus groups
16	Setting	Description of sample	What are the important characteristics of the sample? (e.g., demographic data, date)	Detailed in Table 2 (background, expertise) and Table 3 (demographics)
17	Data collection	Interview guide	Were questions, prompts, guides provided by the	Developed via literature review, refined by expert

			authors? Was it pilot tested?	panel; pilot-tested
18	Data collection	Repeat interviews	Were repeat interviews carried out? If yes, how many?	None reported
19	Data collection	Audio/visual recording	Did the research use audio or visual recording to collect the data?	Audio-recorded focus groups; verbatim transcripts
20	Data collection	Field notes	Were field notes made during and/or after the interview or focus group?	Note-takers for focus groups; NVivo memos for interviews
21	Data collection	Duration	What was the duration of the interviews or focus group?	Interviews ~45–60 min; focus groups ~2 hours
22	Data collection	Data saturation	Was data saturation discussed?	Achieved at 13th interview, confirmed with final 2
23	Data collection	Transcripts returned	Were transcripts returned to participants for comment and/or correction?	Not reported
24	Analysis	Number of data coders	How many data coders coded the data?	At least 2 per transcript; NVivo coding team
25	Analysis	Description of the coding tree	Did authors provide a description of the coding tree?	Themes/subthemes described via thematic analysis stages
26	Analysis	Derivation of themes	Were themes identified in advance or derived from the data?	Combined inductive (interviews/focus groups) and deductive (survey-driven)
27	Analysis	Software	What software, if applicable, was used to manage the data?	NVivo

28	Analysis	Participant checking	Did participants provide feedback on the findings?	Focus groups used as validation and co-interpretation step
29	Findings	Quotations presented	Were participant quotations presented to illustrate the themes/findings? Was each quotation identified?	Illustrative quotes included (e.g., Int_1, Int_7)
30	Findings	Data and findings consistent	Was there consistency between the data presented and the findings?	Constant comparison method used
31	Findings	Clarity of major themes	Were major themes clearly presented in the findings?	Overarching and subthemes presented
32	Findings	Clarity of minor themes	Is there a description of diverse cases or discussion of minor themes?	Contradictory views noted (e.g., online vs in-person training preferences)
