# Supplementary material for: Bridging the AI-Literacy Gap in Health Care: Qualitative Analysis of the Flanders Case Study
Source: J Med Internet Res. 2025 Dec 8;27:e76709. doi: 10.2196/76709 (PMC12685233; doi:10.2196/76709)
Supplement: Checklist 2 [file jmir-v27-e76709-s003.pdf]

CHERRIES Checklist (Checklist for Reporting Results of Internet E-Surveys)

Section	Item	CHERRIES Question	How addressed in the study
Design	IRB approval	Was the study approved by an ethics committee?	Yes – Aristotle University of Thessaloniki, ethics approval 237317/2024
Design	Informed consent	Was informed consent obtained?	Digital opt-in form; anonymity guaranteed
Design	Survey design	Describe survey type, open/closed, voluntary/mandatory	Exploratory, non-probability, voluntary
Development	Pretesting	Was the survey pre-tested?	Pilot with 5 healthcare professionals; minor revisions made
Development	Reliability	Was internal consistency or reliability assessed?	Cronbach's $\alpha = 0.84$ for AI training importance items
Recruitment	Open survey	Was the survey open or closed?	Voluntary participation
Recruitment	Distribution channels	How were participants recruited?	Hospitals, SMEs, alumni lists, VAIA, conferences
Recruitment	Incentives	Were incentives offered?	Optional prize draw; email handled by VAIA staff only
Administration	Platform	Which platform was used?	EU Survey platform
Administration	Duplicate entries	How was multiple entry prevention handled?	Not explicitly reported; system settings prevented multiple

			submissions per session
Administration	Data completeness	How were incomplete questionnaires handled?	Excluded 5 respondents with <20% completion
Response rate	Invitations	How many invitations were sent?	Wide distribution, denominator unknown
Response rate	Responses	How many responses were received?	139 received, 134 valid
Response rate	Population estimate	What was the target population size?	~5,000 healthcare professionals in Flanders
Results	Demographics	Provide demographic breakdown of respondents	Gender, age, occupation, seniority (Table 3)
Results	Missing data	How was missing data handled?	Excluded incomplete responses (<20%)
Results	Analysis	How were data analyzed?	Descriptive statistics, triangulation with interviews/focus groups
Results	Limitations	What are the study's limitations?	Non-probability sampling, modest response rate, limited generalizability
